# Repurposing existing drugs: identification of irreversible IMPDH inhibitors by high-throughput screening

**DOI:** 10.1080/14756366.2018.1540474

**Published:** 2018-11-19

**Authors:** Albertus Eka Yudistira Sarwono, Shinya Mitsuhashi, Mohammad Hazzaz Bin Kabir, Kengo Shigetomi, Tadashi Okada, Fumina Ohsaka, Satoko Otsuguro, Katsumi Maenaka, Makoto Igarashi, Kentaro Kato, Makoto Ubukata

**Affiliations:** a Division of Applied Bioscience, Graduate School of Agriculture, Hokkaido University, Sapporo, Japan;; b Department of Cellular and Molecular Biology, The University of Texas Health Science Center at Tyler, Tyler, TX, USA;; c National Research Center for Protozoan Diseases, Obihiro University of Agriculture and Veterinary Medicine, Obihiro, Japan;; d Center for Research and Education on Drug Discovery, Faculty of Pharmaceutical Sciences, Hokkaido University, Sapporo, Japan;; e Division of Neurology, Respirology and Metabolism, Department of Internal Medicine, Faculty of Medicine, University of Miyazaki, Kiyotake, Miyazaki, Japan

**Keywords:** Drug repurposing, IMP dehydrogenase, irreversible inhibitors, purine metabolic pathway

## Abstract

Inosine 5′-monophosphate dehydrogenase (IMPDH) is an essential enzyme for the production of guanine nucleotides. Disruption of IMPDH activity has been explored as a therapeutic strategy for numerous purposes, such as for anticancer, immunosuppression, antiviral, and antimicrobial therapy. In the present study, we established a luciferase-based high-throughput screening system to identify IMPDH inhibitors from our chemical library of known bioactive small molecules. The screening of 1400 compounds resulted in the discovery of three irreversible inhibitors: disulfiram, bronopol, and ebselen. Each compound has a distinct chemical moiety that differs from other reported IMPDH inhibitors. Further evaluation revealed that these compounds are potent inhibitors of IMPDHs with *k*_on_ values of 0.7 × 10^4^ to 9.3 × 10^4^ M^−1^·s^−1^. Both disulfiram and bronopol exerted similar degree of inhibition to protozoan and mammalian IMPDHs. Ebselen showed an intriguing difference in mode of inhibition for different IMPDHs, with reversible and irreversible inhibition to each *Cryptosporidium parvum* IMPDH and human IMPDH type II, respectively. In the preliminary efficacy experiment against cryptosporidiosis in severe combined immunodeficiency (SCID) mouse, a decrease in the number of oocyst shed was observed upon the oral administration of disulfiram and bronopol, providing an early clinical proof-of-concept for further utilization of these compounds as IMPDH inhibitors.

## Introduction

Inosine 5′-monophosphate dehydrogenase (IMPDH) is an attractive drug target due to its essential role in cellular purine nucleotide biosynthesis. It catalyzes the conversion of IMP to xanthosine monophosphate (XMP), which will be eventually converted to guanosine monophosphate (GMP) for the DNA biosynthesis pathway.

Inhibition of *Cryptosporidium parvum* IMPDH (CpIMPDH) of the pathogenic protozoa *C. parvum* has been proposed as an antiprotozoal therapeutic strategy against the infection. The enzyme plays an important role in the streamline salvage purine nucleotide biosynthesis of the protozoa. Therefore, inhibition of the enzyme resulted in a detrimental effect to the protozoan growth. In human, IMPDH has two isoenzymes, type I and type II. Generally, human IMPDH type I (hIMPDH I) is a ubiquitous enzyme and expressed by various tissues in low level, while hIMPDH II is expressed in a rapidly multiplying cells. Inhibition of hIMPDH II, in particular, has been sought after, due to its role as a chemotherapeutic target for various purposes, such as anticancer, immunosuppressive, and antiviral therapy^1–8^. Therefore, this study was aimed to discover novel inhibitors for CpIMPDH and hIMPDH II, representatives of microbial and mammalian IMPDH, respectively.

In the effort to discover new inhibitors, it is important to not only focusing on novel bioactive compounds but to also in repurposing existing compounds to a novel molecular target. Evidently, repurposing of a known bioactive compound, particularly the ones with established pharmacological properties, could significantly alleviate the intensive labor and enormous financial burden of the conventional drug development processes^9–11^. Furthermore, the introduction of robotics systems into the field of medicinal chemistry, especially as an automatic solution handlings system, has further accelerated the above-mentioned lengthy process. These mechanizations provide scientists with the capability to carry out bioassays with a much higher throughput.

In this study, the discovery and the characterization of three irreversible IMPDH inhibitors: ebselen, disulfiram, and bronopol, were discussed. The inhibition kinetic parameters were then tested for CpIMPDH and hIMPDH II. Overall, this study provided a new perspective of the available classes of irreversible IMPDH inhibitors. The variation in the inhibitor moiety could be beneficial for the future design and development of more potent and selective IMPDH inhibitors.

## Materials and methods

### Molecular methods

The coding sequence of CpIMPDH was amplified by PCR using primer sets 5′-TTTTGGATCCTCAAACATGGGTACA-3′ and 5′-TTTTGAATTCCTATTTACT-ATAATT-3′. The PCR product was cloned into pCR2.1-TOPO vector (Invitrogen Japan KK, Tokyo, Japan), and the complete gene sequence was confirmed using ABI PRISM 3100 Genetic Analyzer (Applied Biosystems, Tokyo, Japan). CpIMPDH gene was digested by *Bam*HI and *Eco*RI, and inserted into a pGEX-6P-2 plasmid (GE Healthcare Bio-Science Corp., Amersham, UK). The plasmid was then transformed to ECOS *E. coli* BL21 (DE3) (Wako Pure Chemical Ind., Ltd., Osaka, Japan).

The plasmid of hIMPDH II was a generous gift from Prof. Lizbeth Hedstrom, Brandeis University, USA. The plasmid was transformed in the same manner as for CpIMPDH.

### Expression and purification of recombinant IMPDH

Cells carrying previously described plasmids were grown overnight at 30 °C in 50 ml 2xYT broth containing 100 μg/ml ampicillin. Then the broth was subcultured to 700 ml of medium containing a final concentration of 1 mM isopropyl-1-thio-*β*-galactopyranoside (Sigma-Aldrich Japan, Tokyo, Japan) and 100 μg/ml ampicillin. After 5 h incubation at 25 °C, cells were harvested by centrifugation, washed with PBS solution, and stored frozen at −80 °C until usage. The cell was later thawed on ice, suspended with 50 mM Tris-HCl pH 8.0 containing 5 mM EDTA (Sigma-Aldrich Japan), 1 mM dithiothreitol (DTT) (Wako Pure Chemical Ind., Ltd.), 0.5 mM phenylmethylsulfonyl fluoride, and 1 μg/ml leupeptin, then sonicated. The cell lysate was centrifuged, the supernatant was applied to Glutathione Sepharose 4B (GE Healthcare Bio-Science Corp.), then mixed gently overnight. The unbound proteins were washed with 30 mM Tris-HCl pH 8.0 containing 1 mM MgCl_2_, 150 mM NaCl, 1 mM DTT, 0.5 mM benzamidine (Sigma-Aldrich Japan), and 2 μg/ml leupeptin. GST-tag was cleaved using PreScission protease (GE Healthcare Bio-Science Corp.) according to manufacturer manual. The enzyme fraction was isolated and dialyzed overnight with 30 mM Tris-HCl buffer (pH 7.5) containing 1 mM MgCl_2_, 1 mM DTT, 0.5 mM benzamidine, and 50% glycerol. All purification processes were carried out at 4 °C unless stated otherwise. The purified IMPDHs were then supplemented with 10 μg/ml leupeptin and stored at −20 °C until usage.

### Establishment and validation of high-throughput screening system

The high-throughput screening (HTS) assay was established using NAD(P)H-Glo Assay Kit (Promega, Madison, WI). Briefly, a substrate solution containing 50 mM Tris-HCl pH 8.0, 200 mM KCl, 0.1 mg/ml BSA, 1.6 mM β-NAD^+^ (Oriental Yeast Co., Tokyo, Japan), 100 μM IMP (Sigma-Aldrich Japan), and assay kit was added to white 384-well plates. Mycophenolic acid (APAC Pharmaceutical LLC, Hangzhou, China), a known IMPDH inhibitor, or compounds from the Pharmakon repositioning library of 1570 off-patent US Food and Drug Administration (FDA)-approved compounds (Microsource Discovery Systems, Inc., CT) were also added to the wells. The reaction was started by addition of enzyme solution containing 50 mM Tris-HCl pH 8.0, 0.1 mg/ml BSA, and CpIMPDH. The reaction was carried out at 30 °C for 30 min in dark. Solution handling was carried out using Mosquito LCP (TTP LabTech Ltd., Melbourn, UK) and Multidrop Combi (Thermo Scientific, Waltham, MA). The production of NADH was monitored by luminescence measurement using either VERITAS Microplate Luminometer (Turner Biosystems, Sunnyvale, CA), or EnSpire Multimode Reader (Perkin-Elmer, Waltham, MA).

The counter assay was carried out by directly incubating assay kit, the library compounds, and an appropriate amount of NADH. The produced chemiluminescence was monitored.

The half maximal inhibitory concentration (IC_50_) values were calculated by plotting the recorded NADH yield against log of compound concentration in GraFit ver. 7 (Erithacus Software, West Sussex, UK). All screening assays in this study was conducted with 0.5% DMSO as vehicle and library compound concentration of 10 μM unless stated otherwise.

### Kinetics of IMPDH inhibition by hit compounds

#### Irreversible inhibition

Standard IMPDH assay solution contains 50 mM Tris-HCl pH 8.0, 100 mM KCl, 3 mM EDTA, 0.1 mg/ml BSA, and an appropriate amount of inhibitor. Substrate concentrations are 500 μM NAD^+^ and 250 μM IMP for CpIMPDH, 100 μM NAD^+^ and 250 μM IMP for hIMPDH II. The reaction was started by the addition of an appropriate amount of enzyme. The activity of the enzyme was measured by monitoring NADH production in absorbance at 340 nm.

The exponential inactivation was quantified by fitting the decay progress curve to Equation (1)^12^^,^^13^:

(1) $$R_{t}-R_{0}=\frac{V_{0}}{k_{\text{obs}}}\left(1-e^{-k_{\text{obs}}.t}\right)$$

where *R*_t_ is the absorbance at time *t*, *R*_0_ is the initial absorbance at time 0, *V*_0_ is the initial reaction rate, and *k*_obs_ is the observed rate constant of enzyme inactivation. Acquired *k*_obs_ values then fitted into Equation (2) to obtain *k*_on_ value:

(2) $$k_{\text{obs}}=\;\frac{k_{\text{on}}\;[I]}{1+\;\frac{\left[S\right]}{K_{m}}}$$

where *k_on_* is the apparent second-order rate constant for IMPDH inactivation, [*I*] is inhibitor concentration, [*S*] is IMP concentration, and *K*_m_ is Michaelis–Menten constant for IMP.

Plot fitting of the inhibitory study was carried out using GraphPad Prism 7.0 (GraphPad Software, San Diego, CA). Values of inhibitory activity were expressed as mean ± SD for two independent experiments.

### Reversible inhibition

Reversible inhibition parameter was measured by monitoring the formation of NADH in various substrate concentrations in the presence and absence of inhibitor. The assay was carried out with standard IMPDH assay solution as mentioned in Irreversible inhibition. Data were fitted into noncompetitive inhibition (Equation (3)) or mixed-model inhibition (Equation (4)):

(3) $$v=\frac{V_{m}[S]}{\left(K_{m}\left(1+\frac{\left[I\right]}{K_{i}}\right)+[S]\left(1+\frac{\left[I\right]}{K_{i}}\right)\right)}$$

(4) $$v=\frac{V_{m}[S]}{\left(K_{m}\left(1+\frac{\left[I\right]}{K_{\text{is}}}\right)+[S]\left(1+\frac{\left[I\right]}{K_{\text{ii}}}\right)\right)}$$

where *v* is reaction velocity, *V*_m_ is the maximal velocity, [S] is substrate concentration, *K*_m_ is the Michaelis–Menten constant for the substrate, *K*_i_ is inhibition constant when the inhibitor shows equal affinity to free enzyme and enzyme-substrate complex, *K*_is_ is the slope inhibition constant, and *K*_ii_ is the intercept inhibition constant.

Plot fitting of the inhibitory study was carried out using GraphPad Prism 7.0. Values of inhibitory activity were expressed as mean ± SD for two independent experiments.

### Enzyme-inhibitor dockings and *C. parvum in vivo* inhibitory studies

Experiment information available as supplementary material.

## Results

### HTS of chemical library compounds

**Figure 1.**
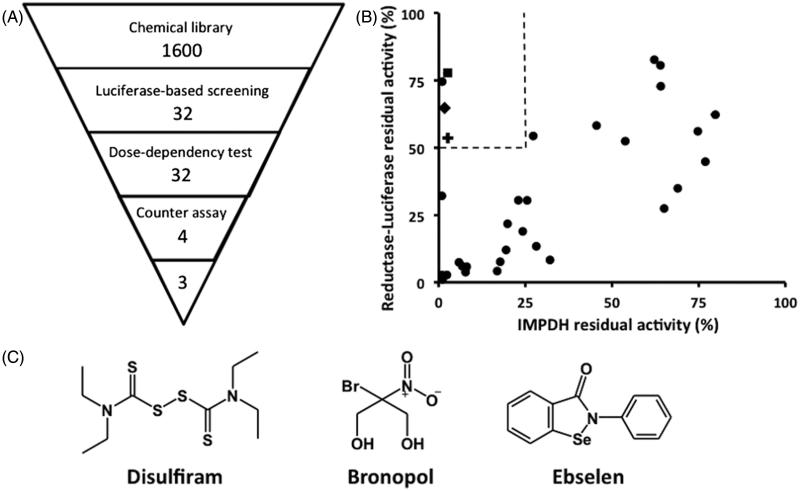
Screening of CpIMPDH inhibitors. (A) Outline of HTS study. Numbers indicate the number of compounds after elimination by each screening step. (B) The counter assay was performed on 32 compounds to exclude reductase–luciferase inhibitors from specific CpIMPDH inhibitors. Dashed line showed the selection criteria, which eliminated inhibitors that inhibit less than 90% of control CpIMPDH activity in HTS assay, while also inhibit more than 50% of control reductase–luciferase activity, in the inhibitor concentration of 10 μM. ▪: disulfiram, ♦: bronopol, O: ebselen. (C) The structures of the hit compounds.

The chemiluminescence-based HTS system enables assays to be carried out with a low microliter volume but still produce robust and selective signal. Signal-to-noise ratio was high, with experimental signal up to 3-magnitudes higher than those of empty wells. The developed system has an average Z’-factor value of 0.7 from three independent experiments (0.71, 0.7, and 0.66), indicating an excellent assay system for screening (Figure S1)^14^. Using this system, a collection of 1400 known bioactive compounds was screened for any CpIMPDH inhibitory activity. The primary screening was conducted with a compound concentration of 10 μM, and resulted in the identification of 32 compounds as hits based on a standard deviation offset. To exclude false-positives, a concentration–response curve test was subsequently carried out. A counter assay as the secondary screening was carried out to exclude any reductase–luciferase inhibitors. Final hits from the screening system comprised of four compounds (0.3%) (Figure 1(A,B)). Finally, three compounds were chosen to be analyzed in this study: ebselen, disulfiram, and bronopol (Figure 1(C)). The fourth compound, thiram – another thiuram disulfide, was excluded from this study.

### The irreversible inhibition of IMPDH by disulfiram and bronopol

The inhibitory activity of the hit compounds was analyzed for both CpIMPDH and hIMPDH II. The characterization of the kinetic parameters of CpIMPDH and hIMPDH II showed similar values to previous reports, with *K*_m_ NAD^+^ of 103 μM and *K*_m_ IMP of 13.7 μM for CpIMPDH and *K*_m_ NAD^+^ 8.9 μM and *K*_m_ IMP of 5.4 μM for hIMPDH II^8^^,^^15^.

**Figure 2.**
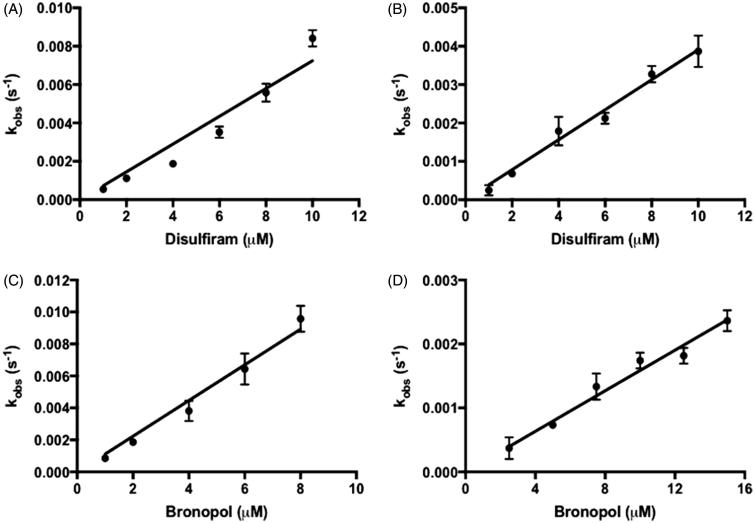
The inactivation of (A) CpIMPDH and (B) hIMPDH II by disulfiram. The inactivation of (C) CpIMPDH and (D) hIMPDH II by bronopol. Values are mean ± SD from two independent experiments.

Determination of the inhibition mode was performed by dialysis, jump dilution, and incubation-time-dependent experiments (data not shown). Disulfiram and bronopol were identified as irreversible inhibitors to CpIMPDH and hIMPDH II. Subsequently, the time-dependent inactivation of IMPDH in the presence of various inhibitor concentrations was fitted into Equation (1) (Figure S2). The acquired *k*_obs_ values were then fitted into Equation (2) (Figure 2). Both compounds were found to be potent inhibitors of IMPDH, compared to other known irreversible IMPDH inhibitors (Table 1). However, both inhibitors also showed a limited selectivity between mammalian and protozoan IMPDH with *k*_on_ CpIMPDH:hIMPDH II value of 0.78 and 3.1 for disulfiram and bronopol, respectively. Inhibition of IMPDH by disulfiram and bronopol showed linear *k*_obs_ plots, which indicated a one-step inactivation mechanism.

**Table 1.** *k*_on_ values of disulfiram, bronopol and ebselen against IMPDHs (M^−1^·s^−1^).

	CpIMPDH	hIMPDH II	*E. coli* IMPDH
Disulfiram^a^	1.4 × 10^4^	1.8 × 10^4^	
Bronopol^a^	2.2 × 10^4^	0.7 × 10^4^	
Ebselen^a^		>9.3 × 10^4^	
EICARMP^b^		1.7 × 10^3^	2.3 × 10^4^
2-VIMP^c^			7.4 × 10^3^
2-FVIMP^d^			2.7 × 10^4^
6-Cl-IMP^e^		44	

a This study. Activity was measured at 30 °C, 50 mM Tris-HCl buffer pH 8.0, 100 mM KCl, 3 mM EDTA, and 0.1 mg/ml BSA. CpIMPDH: 500 μM NAD^+^, 250 μM IMP. hIMPDH II: 100 μM NAD^+^, 250 μM IMP.

b Activity was measured at 25 °C, 50 mM Tris-HCl buffer pH 8.0, 100 mM KCl, 3 mM EDTA, 1 mM DTT. hIMPDH II: 100 μM NAD^+^, 125 μM IMP. *E. coli* IMPDH: 2.5 mM NAD^+^, 1 mM IMP^16^.

c Activity was measured at 25 °C, 50 mM Tris-HCl buffer pH 8.0, 150 mM KCl, 1 mM EDTA, 1 mM DTT. *E. coli* IMPDH: 1.5 mM NAD^+^, 500 μM IMP^17^.

d Activity was measured at 25 °C, 100 mM Tris-HCl buffer pH 8.0, 150 mM KCl, 1 mM EDTA, 1 mM DTT. *E. coli* IMPDH: 2.5 mM NAD^+^, 1 mM IMP^18^.

e Activity was measured at 25 °C, 100 mM Tris-HCl buffer pH 8.0, 100 mM KCl, 3 mM EDTA. hIMPDH II: 400 μM NAD^+^, 200 μM IMP^19^.

The substrate protection experiments showed that the higher concentration of IMP more strongly protect IMPDH from inactivation than higher concentration of NAD^+^, which implies that these inhibitors target the IMP binding site of IMPDH. Molecular docking simulations of disulfiram and bronopol to hIMPDH II crystal provided the prediction of the enzyme–inhibitor interaction in the IMP binding site (Table S1; Figure S4).

### The mixed mode IMPDH inhibition by ebselen

**Figure 3.**
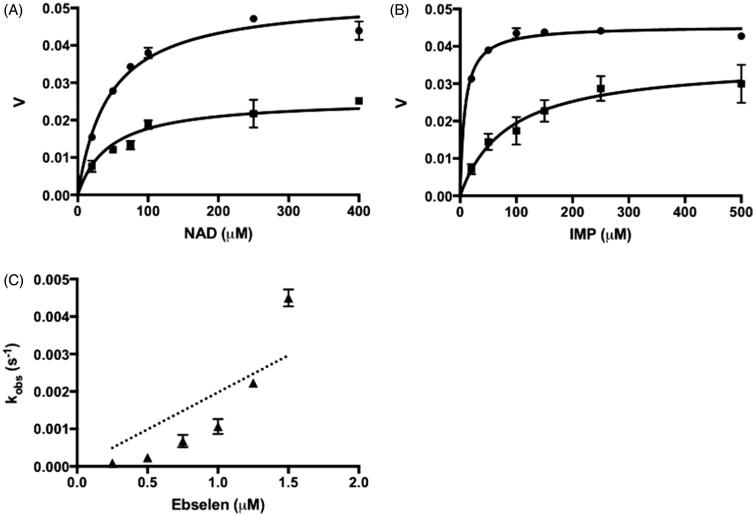
The ebselen inhibition of IMPDHs. (A) Ebselen inhibition in respect to NAD^+^, IMP concentration was kept constant at 250 μM. (B) Ebselen inhibition in respect to IMP, NAD^+^ concentration was kept constant at 400 μM. Circles show the assay with only vehicle and squares show the assay in the presence of 0.75 μM ebselen. Velocity (*v*) is in arbitrary units. (C) Ebselen irreversible inhibition to hIMPDH II. Values are mean ± SD from two independent experiments.

Ebselen showed a quite different inhibition property compared with the other two inhibitors. Ebselen did not exhibit any irreversibility against CpIMPDH, even after decreasing the amount of enzyme and extending the measurement time (Figure S3). Therefore, inhibition of CpIMPDH by ebselen was then treated as a reversible mode. The acquired initial velocity values in the absence and presence of inhibitor were fitted into Equations (3) and (4). Ebselen inhibition of CpIMPDH was found to be noncompetitive in respect to NAD^+^ and mixed in respect to IMP (Figure 3(A,B)). The noncompetitive inhibition against NAD^+^ is a common characteristic of IMP binding site inhibitors. However, mixed-model inhibition in respect to IMP illustrated the complexity of ebselen interaction to the IMP binding site. The fitted *K*_is_ value was much lower than the *K*_ii_ value, indicating a higher affinity of ebselen to free enzymes rather than to enzyme–substrate complexes (Table 2). Taken together, the result indicated that ebselen is a reversible IMP binding site inhibitor of CpIMPDH.

**Table 2.** The inhibition parameters of ebselen against CpIMPDH.

	CpIMPDH
Ebselen (nM)^a^	706 (NC, NAD^+^)
	64.6 ± 16 (*K_is_*, MM, IMP)
	2808 ± 927 (*K_ii_*, MM, IMP)
MPA (nM)^b^	9300 (UC, NAD^+^)
GMP (μM)^b^	46 (C, IMP)

C: competitive; UC: uncompetitive; NC: noncompetitive; MM: mixed-model inhibition.

a This study. Activity was measured at 30 °C, 50 mM Tris-HCl buffer pH 8.0, 100 mM KCl, 3 mM EDTA, and 0.1 mg/ml BSA. Assay against NAD^+^, constant IMP at 250 μM, varied NAD^+^. Assay against IMP, constant NAD^+^ at 500 μM, varied IMP.

b Activity was measured at 25 °C, 50 mM Tris-HCl buffer pH 8.0, 100 mM KCl, 3 mM EDTA, and 1 mM DTT. Assay against NAD^+^, constant IMP at 250 μM, varied NAD^+^ concentration. Assay against IMP, constant NAD^+^ at 500 μM, varied IMP concentration^8^.

In contrary, ebselen showed a potent irreversible inhibition to hIMPDH. Different from the previous inhibitors, the plot of *k*_obs_ versus ebselen concentration was found to be hyperbolic. Forcing this plot into Equation (2) yielded *k*_on_ value of 9.3 × 10^4^ M^−1^·s^−1^ (Figure 3(C); Table 1). Substrate protection experiment and molecular modeling study suggested the higher affinity of ebselen to IMP binding site in the interaction with IMPDH (Table S2; Figure S4).

## Discussions

The discovery of novel irreversible IMPDH inhibitors is very important due to its role as a drug target for the management of chronic conditions such as immunosuppressive, antiviral, and antineoplastic therapy. These therapies might need an extended therapeutic duration that theoretically could be provided by irreversible inhibitors due to its nonequilibrium inhibitory mechanism^16^^,^^17^.

Assigning new targets for known bioactive compounds is a vital process for significantly reducing financial and temporal burden of a drug discovery process. In this study, a HTS system was developed to screen novel inhibitors against IMPDHs from a library of known compounds.

From the screening process, three compounds were identified as irreversible inhibitors of IMPDH: disulfiram, bronopol, and ebselen. Disulfiram has been known as an alcohol aversion drug which acts as inactivator of acetaldehyde dehydrogenase. Intake of disulfiram together with alcohol resulted in the accumulation of acetaldehyde, causing discomforting symptoms thus discouraging further intake^18^. Bronopol was utilized as antiseptic and antifungal compound in aquatic veterinary settings and preservative in cosmetic products^19^^,^^20^. Ebselen has been known as a compound with broad activities, such as anti-inflammatory, anti-atherosclerotic, antibacterial, and anticancer. This compound has also been subjected into clinical trials for acute ischemic stroke and prophylactic neuroprotective^21–27^. Each inhibitor was reported previously as an irreversible inhibitor of cysteine, although with other different target enzymes^19^^,^^20^^,^^28–31^. Therefore, this study is the first report on the compounds as IMPDH inhibitors.

Previously, some research groups have identified several potent irreversible inhibitors, which targeted the unique cysteine residue in the IMP binding site of IMPDH. However, most of the irreversible inhibitors, if not all, are of IMP analogs^32–34^. As the previous inhibitors, the hits from this study also seemed to target the same cysteine residue for their inhibition (Tables S1 and S2). Additionally, pre-incubation of these hit compounds with DTT or GSH before the inhibition assay attenuated their inhibitory activity (data not shown). Taken together, these experiments implied that the inhibition of IMPDHs by these hit compounds was also by cysteine modification. By considering their distinctive structures, this study is also the first reported IMP binding site inhibitors with structures other than IMP analogs^1^^,^^35^.

Disulfiram and bronopol were found to have similar potency and inhibition mechanism in the inhibitory assay. Both exhibited similar high *k*_on_ values, comparable to those of potent irreversible IMPDH inhibitors EICARMP and 2-FVIMP. However, selectivity of disulfiram and bronopol between mammalian and bacterial IMPDH seemed to be weak. Recently, disulfiram has been reported as an anticancer compound by inhibition of NPL4, an adaptor of p97 segregase^36^. As hIMPDH II is also overexpressed in various cancer cells, it is possible that IMPDH inhibition by disulfiram might play a role for the observed antineoplastic activities^1^^,^^37–40^.

Unlike the above two inhibitors, IMPDH inhibition by ebselen was quite intriguing. The mode of IMPDH inhibition by ebselen seemed to be of a mixed-model inhibitory mechanism that changes from a reversible mode to an irreversible mode depending on the concentration of the compound. Incubating hIMPDH II in the presence of ebselen showed the clear decay of enzymatic activity. Interestingly, subsequent *k*_on_ plotting yielded an unconventional hyperbolic curve fit. Lower concentration of inhibitor might have reversible inhibition as the main mode, hence the low *k*_obs_ value, and the irreversible inhibition mode progressively became more dominant as the inhibitor concentration increase. Ebselen begins to irreversibly inactivate hIMPDH II at a concentration around 0.5 μM, and is the most potent irreversible inhibitor of IMPDH reported to date.

As for CpIMPDH, the irreversibility of inhibition was not observed in the respective assay concentration. However, irreversible inhibition was observed in CpIMPDH after the incubation with ebselen in the submillimolar level, and dialysis was unable to restore the activity of the enzyme.

There are at least three possible explanation for this: First, that there are substantial differences of binding sites between IMPDHs in regards to ebselen binding, the second, that ebselen has a critical concentration threshold that triggers the chemical reactivity with cysteine residues if trespassed, and the third, that ebselen is a very slow tight-binding inhibitor of CpIMPDH^13^. To confidently confirm the actual basis of this unique modal alteration, further experiments such as crystallization studies in different ratio of enzyme–inhibitor concentration are necessary. The ambivalence inhibition mode of ebselen has also been reported by other research groups^41–43^. Nevertheless, this study is the first report on the alteration of ebselen inhibition mode from a reversible mode into an irreversible mode in a single dose-dependency plot.

Interestingly, the hit compounds also inhibited guanosine 5′-monophosphate reductase (GMPR) of *T. congolense*, another enzyme in the same purine nucleotide biosynthesis pathway (Figure S5)^44^. Similar to IMPDH, GMPR has also been reported to be dependent on catalytic cysteine for its activity^45^^,^^46^. Therefore, it is possible that the hit compounds act as dual IMPDH-GMPR inhibitors. The decay of enzymatic activity of GMPR was not observed during the time-dependent activity assay, suggesting a reversible inhibition in *T. congolense* GMPR. Inhibition to GMPR is important in order to completely disrupt the salvage purine nucleotide biosynthesis, since GMPR could act as a surrogate enzyme of IMPDH in the presence of ammonia^44^^,^^45^.

The discovery of these irreversible inhibitors has opened the possibility of future utilization of these compounds as chemotherapeutical options for treating various IMPDH-related diseases. As a proof-of-concept experiment, a preliminary efficacy assay of disulfiram and bronopol in *C. parvum*-infected severe combined immunodeficiency (SCID) mice was conducted. It was reported before that inhibitor of CpIMPDH could inhibit the growth of the parasite^47^. Both disulfiram and bronopol showed a clear inhibitory activity to *C. parvum* growth with 48 and 30% decrease of parasite burden compared to the control group, respectively. Bronopol, however, showed significant toxicity, and one mouse died in the day 5 of the experiment (Figure S6). In other reports, selenium and ebselen have also been found to effectively inhibit the growth of *C. parvum*^48^^,^^49^. Other experiments are currently ongoing to validate this result. Nevertheless, these results have forecasted the possibility of these hit compounds to be utilized as chemotherapeutic agents for other IMPDH-dependent diseases. To support this proposed utilization, further study is necessary to determine IMPDH resynthesis rate in various therapeutic conditions.

In conclusion, three known compounds have been repurposed as irreversible IMPDH inhibitors: disulfiram, bronopol, and ebselen. The compounds potently inhibit both mammalian and microbial IMPDH in this study, thus could be utilized in various therapeutic settings upon optimization of effective *in vivo* dosing. The variety of reactive moieties in these compounds certainly provides beneficial information for future structure–activity relationship study of IMP binding site inhibitors. While the concentration of disulfiram and bronopol used in the preliminary *in vivo* anti-cryptosporidiosis experiment was high, the positive results suggest that the inhibitors could also be explored as antimicrobial agents in combinatorial therapy in a lower concentration, especially for targeting GSH-lacking Gram-positive bacteria such as *H. pylori*, *M. tuberculosis*, *S. aureus*, *B. subtilis*^50^^,^^51^. However, the efficacy of the hit compounds should also be evaluated for other therapeutic indications of IMPDH inhibitors, such as anticancer, immunosuppression, and antiviral therapy. Special care should be given to bronopol, which showed significant toxicity in the *in vivo* assay. Characterization of other IMPDH inhibitors from our novel compound library and more comprehensive *in vivo* study are currently ongoing.

## Supplementary Material

New-Revised_Supplementary_Material_HTS_of_IMPDH_inhibitors_-_J._Enzy._Inhibit_MU-AS-MU-AS_.docx
