# Supplementary material for: Repurposing existing drugs: identification of irreversible IMPDH inhibitors by high-throughput screening
Source: J Enzyme Inhib Med Chem. 2018 Nov 19;34(1):171–8. doi: 10.1080/14756366.2018.1540474 (PMC6249553; doi:10.1080/14756366.2018.1540474)
Supplement: New-Revised_Supplementary_Material_HTS_of_IMPDH_inhibitors_-_J._Enzy._Inhibit_MU-AS-MU-AS_.docx [file IENZ_A_1540474_SM8066.docx]

**Supplementary Material**

**Repurposing Existing Drugs: Identification of Irreversible IMPDH Inhibitors by High-Throughput Screening**

Albertus Eka Yudistira Sarwono^1^, Shinya Mitsuhashi^1,2^, Md. Hazzaz Bin Kabir^3^, Kengo Shigetomi^1^, Tadashi Okada^3,5^, Fumina Ohsaka^4^, Satoko Otsuguro^4^, Katsumi Maenaka^4^, Makoto Igarashi^3^, Kentaro Kato^3^, and Makoto Ubukata^1^

^1^Division of Applied Bioscience, Graduate School of Agriculture, Hokkaido University, Kita-ku, Sapporo, Hokkaido 060-8589, Japan

^2^Department of Cellular and Molecular Biology, The University of Texas Health Science Center at Tyler, Tyler, Texas 75708, USA

^3^National Research Center for Protozoan Diseases, Obihiro University of Agriculture and Veterinary Medicine, Inada, Obihiro, Hokkaido 080-8555, Japan

^4^Center for Research and Education on Drug Discovery, Faculty of Pharmaceutical Sciences, Hokkaido University, Sapporo 060-0812, Japan

^5^Division of Neurology, Resoirology, Endocrinology and Metabolism, Department of Internal Medicine, Faculty of Medicine, University of Miyazaki, 5200 Kihara, Kiyotake, Miyazaki, 889-1692, Japan

1. **Validation of HTS against IMPDH. Fig. S1.**
2. **Calculation of *k*_obs_ for IMPDH irreversible inhibition. Fig. S2**
3. **Reversibility of ebselen inhibition against CpIMPDH. Fig. S3**
4. **Computational docking study of hit compounds. Fig. S4**
5. **Inhibition of hit compounds to *T. congolense* GMPR. Fig. S5**
6. **Inhibition of *C. parvum* growth *in vivo* by disulfiram and bronopol. Fig. S5**
7. **Substrate protection against disulfiram, bronopol, and ebselen in IMPDH. Table S1 and S2**
8. **Validation of HTS against IMPDH**

To establish z’-factor of the HTS model, 100 nl of 200 mM MPA (positive control) or DMSO (vehicle) was added to 384-wells plate with Mosquito LCP. Subsequently, 10 μl of reaction solution and 10 μl of start solution were added to each wells with Multidrop Combi. Incubation was carried out at 30°C, 30 minutes, in dark.

Z’-factor value is defined as: Z’-factor =1-[(3σc^-^ + 3σc^+^)/(μc^-^ - μc^+^)]; where σc^-^ and μc^-^ denote standard deviation and mean signal value of assay samples with only vehicle, respectively, and σc^+^ and μc^+^ each denotes standard deviation and mean signal value of assay samples with reference inhibitors.


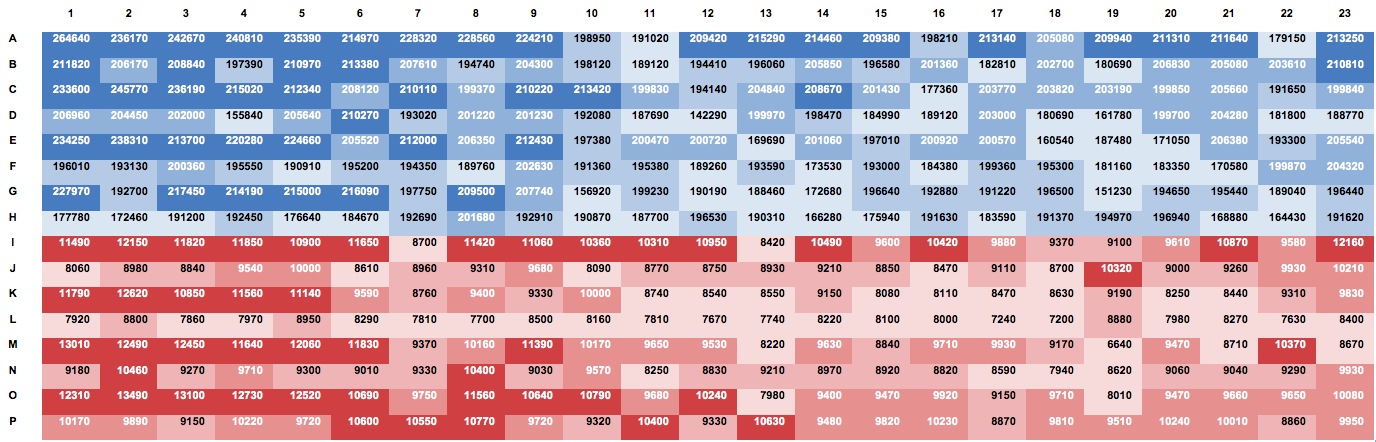

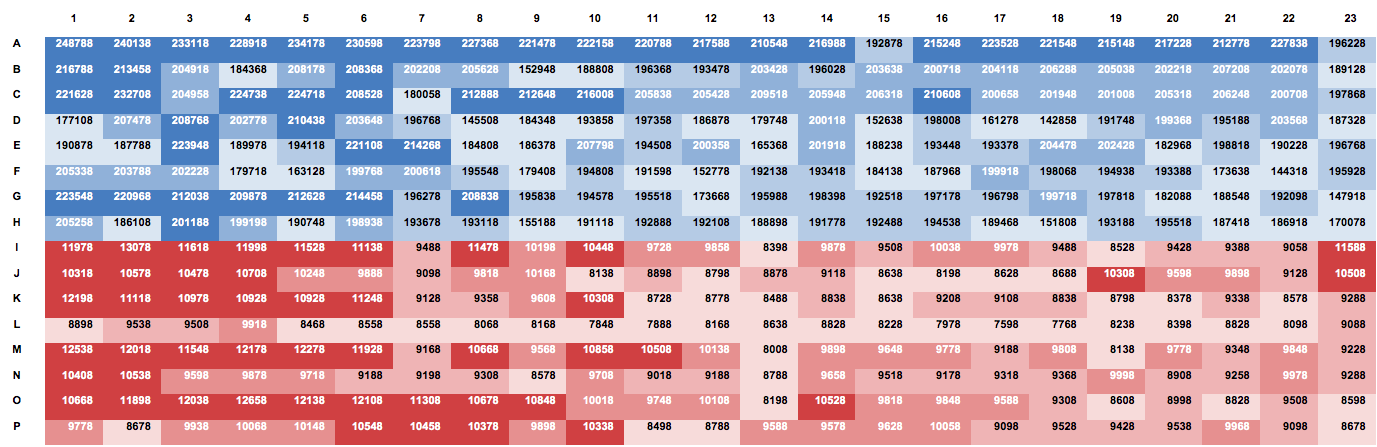

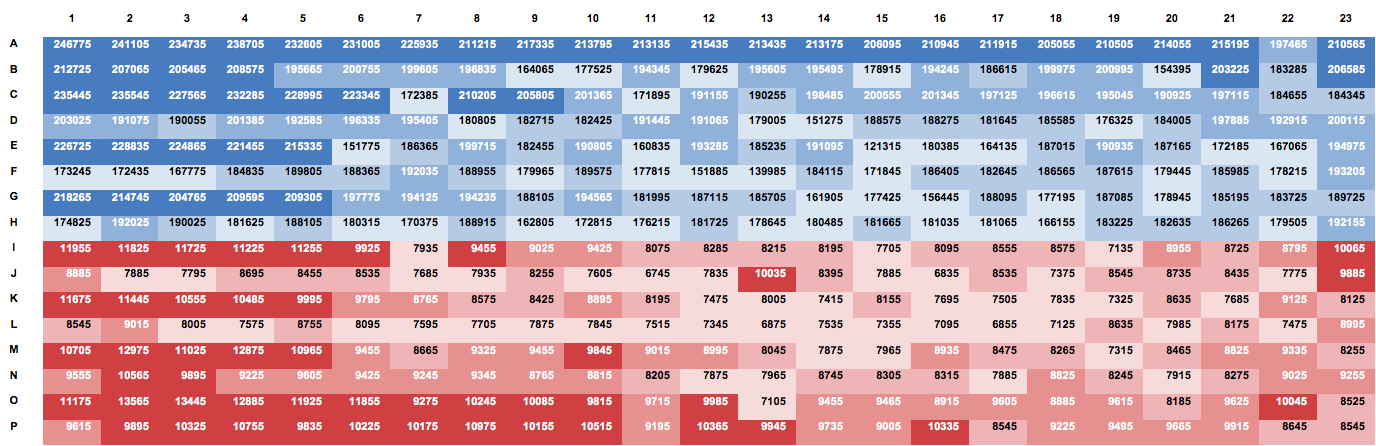

**Fig. S1.** Z’-factor calculation

1. **Calculation of *k*_obs_ for IMPDH irreversible inhibition**

Values *k*_obs_ were acquired by quantification of enzyme exponential decay in the presence of inhibitor compounds (Eq. 1). One replicate of each experiment was presented as representatives.

**Fig. S2.** *k*_obs_ experimental data. Each figure is disulfiram inhibition against CpIMPDH (A) and hIMPDH II (B), bronopol inhibition against CpIMPDH (C) and hIMPDH (D), and ebselen inhibition against hIMPDH II (E).

1. **Reversibility of ebselen inhibition against CpIMPDH**

**Fig. S3.** Ebselen reversibly inhibit CpIMPDH. No exponential enzyme decay observed even after reducing enzyme concentration for 500 times and elongating the reaction time for 9 times. Assay was carried out with standard IMPDH assay solution described in Material and Method.

1. **Computational docking study of hit compounds**

**Preparation of small molecule ligands**

Structures of hit compounds were prepared with SPARTAN ‘16 (Wavefunction, Inc., Irvine, CA, USA). Briefly, molecules were constructed and optimized with Molecular Mechanics MMFF mode. Twenty conformers with the lowest energy were selected for next optimizations. Next, the energy of the conformers was calculated by Density Functional ωB97X-D, 6-31G* mode. Ten conformers with the lowest energy were selected. Lastly, the conformers were subjected to Equilibrium Geometry, Semi-Empirical PM3 mode, and the conformer with the lowest energy was saved for further experiment.

**Preparation of protein**

The x-ray crystal of hIMPDH II protein model (accession code: 1NFB) was prepared with BIOVIA Discovery Studio 2017 R2 (Dassault Systemes Biovia Corp., San Diego, CA, USA) and AutoDock Tools (The Scripps Research Institute, La Jolla, CA, USA). Chain A of the dimer protein was used for the experiment. Water molecule and ligands were deleted, and polar hydrogen was added.

**Molecular docking and visualization**

Molecular docking was performed with Autodock Vina (The Scripps Research Institute, La Jolla, CA, USA). Docking configuration was set as follows to include both binding sites of IMPDH:

Center x, y, and z are each 87.962, 46.668, and 4.916, respectively. Size of x, y, and z-axis are each 38, 28, 36, respectively.

Binding models that showed rational protein-ligand interaction was selected. Result visualization was generated with PyMOL (Schrödinger, LLC) and LigPlus (EMBL-EBI, Cambridge, UK).

**Fig. S4.** The docking model of hit compounds disulfiram (A), bronopol (B), and ebselen (C) to hIMPDH II.

1.
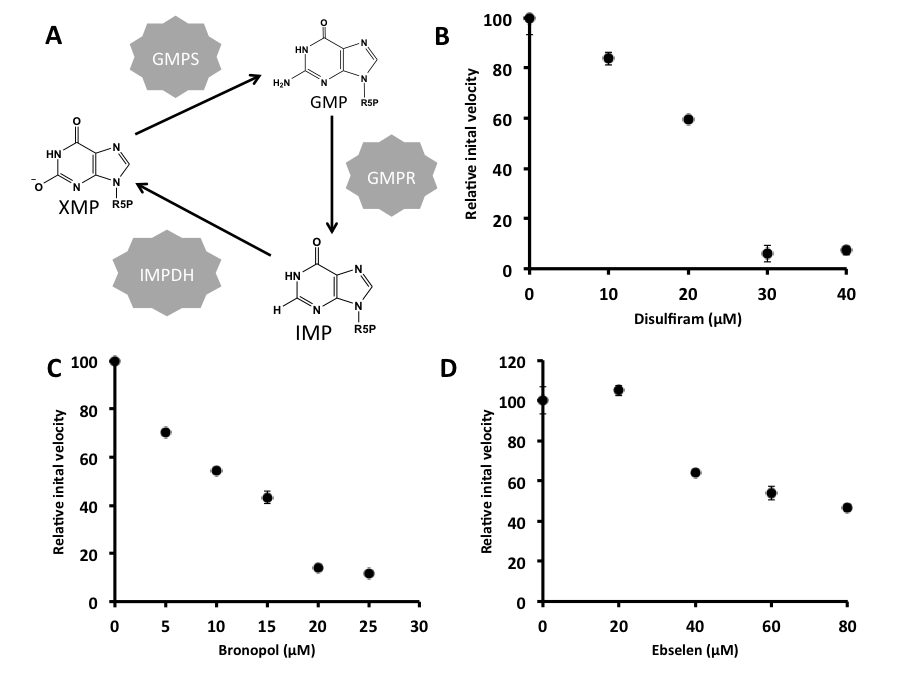
**Inhibition of hit compounds to *T. congolense* GMPR**

**Fig. S5.** The regulation of purine nucleotides by three enzymes: IMPDH, GMP synthase, and GMPR (A). IMPDH inhibitor disulfiram (B), bronopol (C), and ebselen (D) also inhibit *T. congolense* GMPR activity. GMPR inhibition assay was carried out in 75 mM Tris-HCl pH 8.0, 100 mM KCl, 3 mM EDTA, 0.1 mg/ml BSA, 100 μM NADPH and 350 μM GMP. Reaction was started with addition of appropriate amount of enzyme. The activity of the enzyme was measured by monitoring NADPH consumption at absorbance of 340 nm.

1. **Inhibition of *C. parvum* growth *in vivo* by disulfiram and bronopol**
   Nine adult (4 weeks-old) SCID mice were divided into three groups: three mice in each group of disulfiram treatment, bronopol treatment, and control. 1×10^5^ of purified *C. parvum* oocysts were inoculated orally by gavage in each mouse. Experimental concentration of disulfiram and bronopol were 1,000 mg/kg and 20 mg/kg body weight per day, respectively. The drug was orally administered once daily for 2 weeks, with the first administration carried out 4-hours post infection. Distilled water was administered as control.
   The weight of each mouse was measured daily, and amount of administered drug was adjusted accordingly. The number of oocysts in feces was counted under microscope by sucrose gradient floatation method for 2 weeks.

The mice were housed individually in cages with wire-mesh floor and maintained on standard laboratory feed pellets and water *ad libitum*. All animal procedures were approved by the ethics committee on the use of animals in Obihiro University of Agriculture and Veterinary Medicine (approval No. 29-72).

**Fig. S6.** The time course of *C. parvum* growth inhibition by hit compounds (◼) disulfiram (A) and bronopol (B) compared to negative control (🞆) in a mouse model. Each data was the average value from 3 mice. The exception is only for bronopol group, where one mouse died at day 5th (*).

1. **Substrate protection against disulfiram, bronopol, and ebselen in IMPDH**

**Table S1.**

Substrate protection against disulfiram and bronopol in CpIMPDH

| IMP (μM) | NAD^+^ (μM) | *k*_obs_ (.10^-4^ s^-1^) | |
| --- | --- | --- | --- |
|  |  | Disulfiram | Bronopol |
| 250 | 500 | 20 | 20 |
| 500 | 500 | 11 | 9 |
| 1000 | 500 | 7 | 6 |
| 1500 | 500 | 5 | 5 |
| 250 | 250 | 25 | 17 |
| 250 | 500 | 19 | 13 |
| 250 | 1000 | 15 | 10 |
| 250 | 1500 | 13 | 8 |

Assay was carried out with standard IMPDH assay solution described in Material and Method. NADH formation was measured by monitoring emission at 465 nm (excitation 340 nm). The value of *k*_obs_ was measured by fitting the progress of the reaction into Eq. 1.

**Table S2.**

Substrate protection against ebselen in hIMPDH II

| IMP (μM) | NAD^+^ (μM) | *k*_obs_ (.10^-4^ s^-1^) |
| --- | --- | --- |
| 50 | 300 | 8 |
| 100 | 300 | 6 |
| 150 | 300 | 4 |
| 300 | 300 | 2 |
| 300 | 50 | 5 |
| 300 | 100 | 4 |
| 300 | 150 | 3 |
| 300 | 300 | 2 |

Assay was carried out with standard IMPDH assay solution described in Material and Method. NADH formation was measured by monitoring emission at 465 nm (excitation 340 nm). The value of *k*_obs_ was measured by fitting the progress of the reaction into Eq. 1.
